# Discomfort relief after paracetamol administration in febrile children admitted to a third level paediatric emergency department

**DOI:** 10.3389/fped.2023.1075449

**Published:** 2023-03-09

**Authors:** Elena Chiappini, Matilde Bestetti, Stefano Masi, Teresa Paba, Elisabetta Venturini, Luisa Galli

**Affiliations:** ^1^ Pediatric Infectious Disease Unit, Anna Meyer Children's University Hospital, Florence, Italy; ^2^ Department of Health Sciences, University of Florence, Florence, Italy; ^3^ Department of Emergency Medicine, Anna Meyer Children's University Hospital, Florence, Italy

**Keywords:** discomfort, febrile children, fever, paracetamol, Acetaminophen

## Abstract

**Background:**

international guidelines recommend treating fever in children not at a predefined body temperature limit but based on the presence of discomfort. However few studies evaluated discomfort relief after administration of antipyretics in children.

**Methods:**

Between 1st January and 30th September 2021 a single-center prospective observational study was performed in febrile children consecutively admitted to a pediatric emergency department and treated with paracetamol orally. For each child, body temperature, presence and severity of discomfort, defined using a previously published semiquantitative likert scale, were evaluated at baseline and 60 min after administration of paracetamol, and differences were analyzed.

**Results:**

172 children (males: 91/172; 52.9%; median age: 41.7 months) were included. Significant reductions in body temperature (median body temperature at T0: 38.9 °C; IQR: 38.3–39.4, median body temperature at T60: 36.9 °C; IQR: 36.4–37.5; *P* < 0.0001), and in the level of discomfort (proportion of children with severe discomfort at T0: 85% and at T60:14%; *P* < 0.0001) were observed. Severe discomfort at T60 persisted in a minority of children (24/172; 14%) and it was not related to body temperature values.

**Conclusions:**

paracetamol in febrile children is associated not only with significantly reduction in body temperature but also with discomfort relief.

## 1. Introduction

Several guidelines are available for the management of fever in children in different areas of the world (1). One of the key messages shared by the majority of guidelines is to treat fever not at a predefined body temperature limit but basing on the presence of discomfort (1).

However, according to previously published data, less than 50% of pediatricians follow this recommendation (2). One possible explanation of this finding could be the lack of a univocal definition of discomfort and the fact that the assessment of discomfort itself can be difficult to be performed, particularly in children (2). Evaluation on discomfort modification over time is also challenging since quantitative assessment of discomfort is complex and depends on subjective factors such as the age of the child, his ability to communicate his state of discomfort, and the characteristics of the adult assessing the child. Socio-cultural factors and the presence and degree of fever-phobia can also influence the tutor's interpretation of the child's discomfort (2).

A recent Italian consensus conference, conducted in order to obtain a definition of discomfort, to improve the management of feverish children and to promote adherence to guidelines recommendations (3), considered variations of sleep-wake cycle, appetite, motor activity, mood, daily habits, and facial expression. Using this previously published tool, the presence and modification of discomfort in febrile children admitted to pediatric emergency room at baseline (T0) and 60 min after oral paracetamol administration (T60) was evaluated.

## 2. Materials and methods

### 2.1. Study aims

The aim of this study was to assess the degree of discomfort and its alterations before and one hour after the administration of paracetamol in febrile children (aged >28 days), attending to the Emergency Department of the Meyer Children's University Hospital, Florence between 1st January and 30th September 2021.

### 2.2. Definitions

Fever was defined as an increase in body temperature values above normal levels (1). From a practical point of view, for the purposes of this study, children with an auricular temperature above 37.5 °C were considered feverish (1).

Discomfort was defined on the basis of a previously published study (3). Briefly the tool in Supplementary Appendix S1 was used to evaluate it, considering the presence and grade in different items including variations of sleep-wake cycle, appetite, motor activity, mood, daily habits, and facial expression. Severe discomfort was defined as a number of altered items greater than 8.

### 2.3. Study design

A single-center prospective observational study was conducted in febrile children admitted to Emergency Department of the Meyer Children's University Hospital, Florence and treated with paracetamol. The hospital is a pediatric university hospital, and a third level hospital. Every year 44,000 children are admitted to the emergency department and 150 beds are available for hospitalization. All children were consecutively admitted between 1st January 2021 to 30th September 2021, the enrolment was stopped when the sample size of 200 children was obtained. At baseline, the body temperature of each child was measured by appropriately trained nurses, using a tympanic ear thermometer and in compliance with the procedures routinely in place at the same facility (1).

In the same hospital, following internal operating protocols, feverish children receive a dose of antipyretic orally, usually paracetamol (in syrup or drops), administered by the nursing staff, following the prescription by the Emergency Department pediatricians. In accordance with the national guidelines (1), the standard dose of paracetamol is 15 mg/kg every 6 h, up to a maximum of 60 mg/kg/day. In infants up to three months, a dose of 10 mg/kg is administered, up to a maximum of 40 mg/kg/day.

Since this was an observational study, there was no interference by the investigators in the choice of whether or not to administer the antipyretic and the type of drug, dosage or method of administration.

This procedure did not change the length of time the patient had to wait in the emergency department and did not affect the child's diagnostic and therapeutic procedure, as the items collected (weight, age, heart rate, respiratory rate and body temperature) were already routinely assessed by the triage nurses in all children attending the emergency department.

### 2.4. Study population

#### 2.4.1. Children included in the study

Children consecutively admitted to the pediatric the Emergency Department of the Meyer Children's University Hospital, Florence, aged between 29 days and 18 years, with fever who were given oral paracetamol were prospectively enrolled in the study.

#### 2.4.2. Exclusion criteria

Children under the age of 28 days and children with chronic diseases of any kind (e.g., neurological, hematological, oncological, rheumatological conditions) were excluded from the study, as children with these conditions may have a general background of discomfort that could make the study more difficult. Children who were not given any oral antipyretics, those who were given intravenous paracetamol or an antipyretic drug other than paracetamol, such as ibuprofen, were also excluded.

Moreover, children were excluded if, at the time of observation, procedures that might have influenced the assessment of discomfort (e.g.,: taking blood samples, administering inhalation therapy by aerosol, intravenous, oral or intramuscular therapy, applying x-rays or other similar interventions), were being performed.

### 2.5. Data collection

The level of discomfort of each child enrolled was assessed at time zero (T0), before the administration of paracetamol, and at 60 min (T60). In particular T0 was considered as the time at which children arrive in triage, where after an initial assessment by nursing staff if they were feverish, they received an antipyretic, under prescription.

The tool for assessing discomfort was based on a grid (Supplementary Appendix S1) of items which are defined by a panel of Italian experts (4) and included in Table 1. A semi-quantitative assessment was carried out using a 10-point likert scale, similar to a previous study (5). At the two time intervals (T0 and T60) the level of pain was also assessed using an age-appropriate pain scale (6), the *Faces Pain Scale-Revised* (FPS-R) (7). This scale includes six facial expressions with a score ranging from 0 (no pain) to 10 (maximum pain), increasing the score by 2 for each expression (8).

**Table 1:** Signs of discomfort in a feverish child.

**Signals of Discomfort**	Variations of Sleep-Wake Cycle	Delayed sleep phase
		Early sleep phase
		Night awakenings
	Appetite Variations	Reduced appetite
		Reduced liquid intake
	Variation in Motor Activity	Increased complaining
		Restlessness
		Weakness
		Fatigue
	Change in Mood	Irritability
		Anger
		Weeping
	Variation in Daily Habits	Play reduction
		Reduction in showing interests
		Seeking comfort
		Uncooperative
	Variation of Facial Expression	Changed look
		Clenched teeth
		Curled lips
		Wrinkled forehead
		Paleness/colour change
	Other Signals	Tachypnoea
		Chills
		Widespread pain

The assessment of the other items was carried out by direct observation of the investigator and completed by interviewing the parents/caregivers in consideration of their age and cooperation.

The evaluation was carried out every day of the week during daytime hours, between 8:00 a.m. and 8:00 p.m.

The following information was collected anonymously for each child and entered into a special electronic database: age, sex, ethnicity, place of birth, place of origin of the parents/caregivers, body temperature at admission, body temperature at 60 min after administration of paracetamol, drugs administered in the previous 12 h, respiratory rate, heart rate, discomfort items at admission and at T60, type, dosage and method of administration of paracetamol. The data was then entered into an electronic database in anonymous form.

### 2.6. Statistical analysis

Categorical variables were expressed as number and percentages (%), and continuous variables were expressed as median values and interquartile ranges (IQRs). Fisher's exact test or chi-square test was used to compare categorical variables, as appropriate. For continuous variables, differences between groups were analyzed using the Mann-Whitney U-test for non-parametric distributions.

The presence of a possible linear correlation between some continuous variables (e.g., body temperature at T0–T60, number of altered items and pain scale values) was explored using the linear regression test, calculating *R*^2^ and *P*. The Kruskall-Wallis test was used to assess differences between population groups (e.g., age classes, number of altered items grouped into classes) and continuous variables such as body temperature at T0 and T60, and *P*-values were calculated.

The data was analyzed using SPSS Statistics for Windows, Version 27 (IBM Corp., Armonk, NY, USA). *P*-value <0.05 was considered statistically significant.

#### 2.6.1. Size of the sample and power of the study

Calculating the power of the study, given a type I error of 0.05% and a power of 80%, allowed 85 patients per group to be calculated as needed in order to detect a 30% difference in the proportion of children with altered discomfort items at T60, from a proportion of 70% at T0.

#### 2.6.2. Ethics committee and funding

The study was approved by the Ethics Committee of the Meyer Children's Hospital, with the code PSP-FEB and register number 250/2020 on 06/10/2020. All parents or guardians gave written informed consent for the participation of each child before the beginning of the study, according to the Italian legislation.

## 3. Results

Overall, 200 children were enrolled, everyone was febrile when admitted at triage, 28 were excluded because of the exclusion criteria, 172 were included in this study (Males: 91/172; 52.9%; median age: 41.7 months; IQR: 12–48; median body temperature at T0: 38.9 °C; IQR: 38.3–39.4; median body temperature at T60: 36.9 °C; IQR: 36.4–37.5). The difference in body temperature between T0 and T60 was statistically significant (*P* < 0.0001). No adverse event to paracetamol were reported during the study period.

The demographic and clinical characteristics of the children are reported in Table 2.

**Table 2:** Demographic and clinical characteristics of the 172 children included in the study.

Characteristic/variable	Children
**Sex**	***n*; %**
M	91; 53%
F	81; 47%
**Age (month)**	***n*; %**
0–24	110; 64%
25–36	13; 8%
>37	49;28%
Median	24
IQR	12–48
**Ethnic group**	***n*; %**
Caucasian	146; 85%
Afro-American	6; 3%
Asian	13; 8%
South-American	7; 4%
**Place of birth**	***n*; %**
Italy	169; 98%
Foreign	3; 2%
**Origins of parents**	***n*; %**
Italy	132; 77%
Foreign	40; 23%
**Body temperature upon admission °C**	
Median (°C)	38.9
IQR (°C)	38.3–39.4
**Body temperature after 1 h °C**	
Median (°C)	36.9
IQR (°C)	36.4–37.5
**Variation of body temperature °C**	***n*; %**
0–1 degree	23; 13%
1–2 degree	85; 49%
2–3 degree	43; 25%
>3 degree	21; 12%
Median (degree)	1.9
IQR (degree)	1.3–2.4
**Children with/without fever**	***n*; %**
Without fever	85; 91%
With fever	15; 9%
**Admission diagnosis**	***n*; %**
Fever	138; 80%
Abdominal pain	10; 6%
Convulsion	7; 4%
Nausea/vomit	7; 4%
Headache	3; 2%
Dyspnea	3; 2%
Otolaryngological disorder	1; 1%
Ophtalmic disorder	1; 1%
Suspected PIMPS^a^	1; 1%
Stomatitis	1; 1%
**Discharge dimission**	***n*; %**
Viral infection/Infection of the upper airways	96; 56%
Urinary tract infection	17; 10%
Convulsion	8; 8%
Gastroenteritis	9; 6%
Acute otitis media	7; 4%
Appendicopathy	6; 3%
SARS-CoV-2 infection	5; 3%
Mesenteric Lymphadenitis	3; 2%
Stomatis	3; 2%
Pneumonia	3; 2%
Abdominal pain	2; 1%
Perianal abscesses	2; 1%
PIMS^a^	2; 1%
Others	9; 9%
**Outcome**	***n*; %**
Discharge	101; 59%
Brief intensive observation (BIO)	36; 21%
Hospitalisation	35; 20%

^a^ PIMS: SARS-Cov-2 related paediatric multisystem inflammation syndrome.

*Assessment of the degree of discomfort at T0 and T60 following paracetamol administration*.

**Figure 1:**
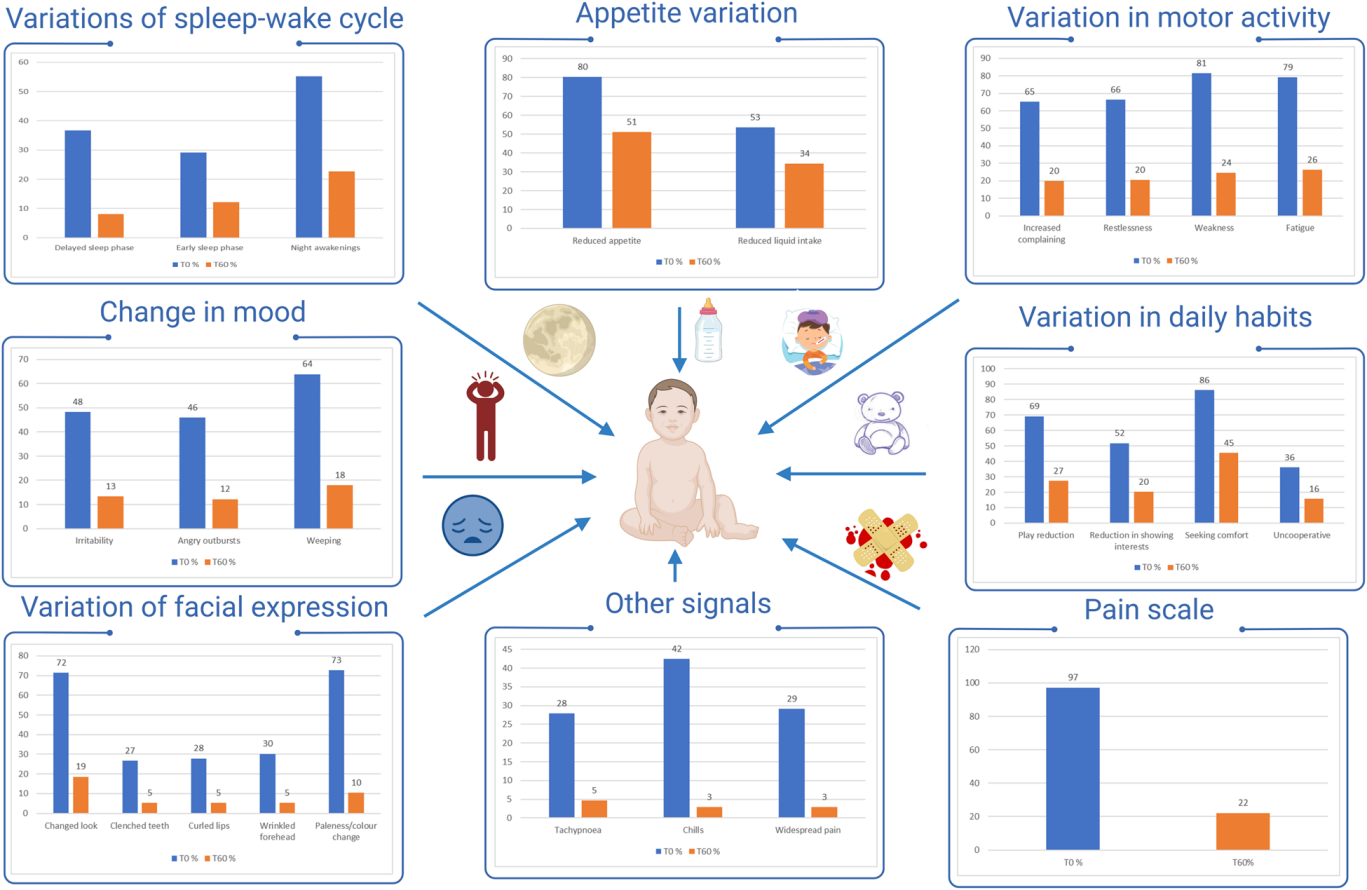
Proportion of studied children with altered items. *% altered parameters T*0 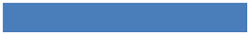, *% altered parameters T*60 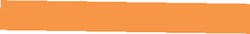.

As summarized in Figure 1, the most frequently altered items at T0 were those related to appetite (reduced appetite: 80%; 138/172), motor activity (increased complain: 65%; 114/172, restlessness: 66%; 114/172, weakness: 81%;140/172, fatigue: 79%; 136/172), mood (weeping: 64%; 110/172), daily habits (play reduction: 69%; 119/172, seeking comfort: 86%; 148/172) and facial expression (changed look: 72%; 123/172).

For all items, a significant change in the proportion of children with altered items was observed between T0 and T60 (Figure 1). At T60 only seeking comfort (45%; 78/172) and reduced appetite (51%; 88/172) were still altered in a substantial proportion of children.

A more pronounced decrease was observed for the items: increased complain and restlessness (T0:65%–66%; T60: 19%–20%), weakness (T0: 81%; T60: 24%), fatigue (T0: 79%; T60: 26%), anger (T0: 46%; T60: 12%), weeping (T0: 64%; T60: 18%), play reduction (T0: 69%; T60: 27%), changed look (T0: 72%; T60: 19%).

With regard to pain assessment at T0, most children had a value equal to or greater than 6 on the pain scale (96%, 165/172), whereas at T60, 78% (133/172) children had a normal or slightly impaired value on the pain scale (0, 2, 4), and 37% showed values of 0–2 (*P* < 0.0001, *Y*^2^ = 73.8) (Figure 1).

**Figure 2:**
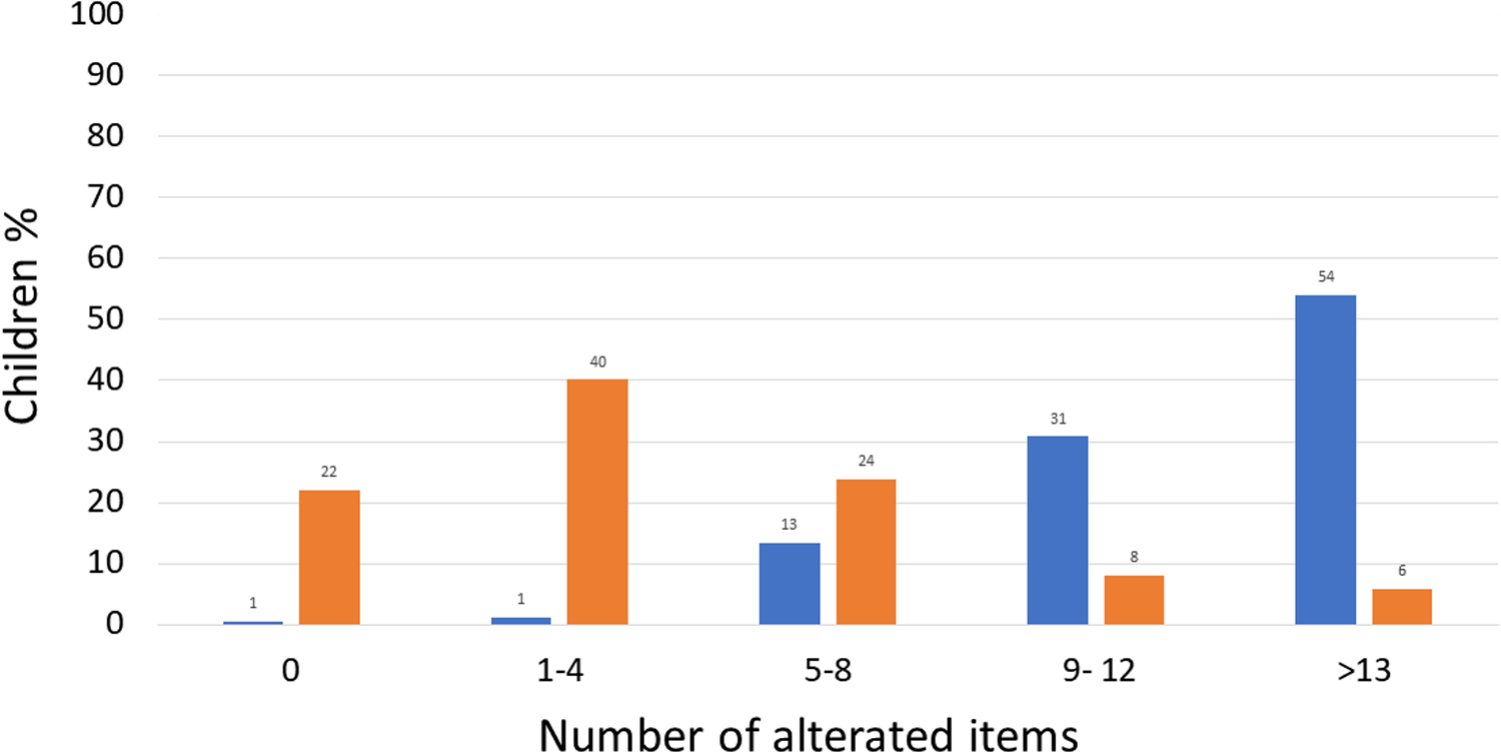
Number of altered items at T0 and T60 in studied children. *T*0 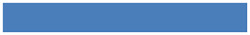 *T*60 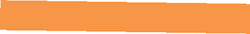.

Assessing the number of impaired items, at T0, 54% of children (93/172) had more than 13 impaired items, 31% (53/172) between 9 and 12, 13% (23/172) between 5 and 8, 1% (2/172) between 1% and 4% and 1% (1/172) had 0 altered items as illustrated in Figure 2.

A significant reduction (*P* < 0.00001, *Y*^2^ = 144.5) was observed at T60 in the proportion of children with more than 13 altered items (6%; 10/172) and between 9 and 12 altered items (8%; 14/172), while the proportion of children with 0 altered items increased (22%; 38/172), 1–4 (40%; 69/172), and 5–8 (24%; 41/172).

As reported in Supplementary Appendix S2, which shows the differences between the demographic and clinical characteristics of children with severe and less severe discomfort, no significant differences between the two groups according to age, sex, body temperature at T0 and T60, reason for hospitalization and outcome.

The group of children that are still feverish at T60 comprised children with a significantly higher median body temperature upon admission than the group of children without fever at T0 (*P* < 0.001) and with a smaller drop in body temperature at T60 (*P* = 0.012), but in whom a significant reduction in median body temperature was still observed (39.9 °C IQR:39.3–40.0 °C vs. 38.0 °C IQR:38.0–38.3; *P* < 0.0001) (Supplementary Appendix S3).

This group of children also had a greater degree of discomfort at T0 (87.3% vs. 60.0% with severe discomfort, more than 8 altered items; *P* = 0.005) but not at T60 (15.3% vs. 0% *P* = 0.134) than the children without fever.

Studying the correlation between the different item, as shown in Supplementary Appendix S4, a significant linear correlation between the number of altered items at T0 and T60 was observed (*P* < 0.001; *R*^2 ^= 0.200), as well as between the body temperature at T0 and T60 (*P* < 0.001; *R*^2 ^= 0.171). By contrast, there was no significant correlation between body temperature at T0 and the number of altered items at T0 (*P* = 0.641; *R*^2^ = 0.001), between body temperature at T60 and the number of altered items at T60 (*P* = 0.916; *R*^2^ = 6.499) and in the difference between the number of altered items at T0 and T60 and the temperature at T0 and T60 (*P* = 0.722; *R*^2 ^= 7.455).

No differences were observed between the two groups according to age, sex, reason for admission and outcome of admission (Supplementary Appendix S3).

Considering pain evaluation (Supplementary Appendix S5), no significant correlation was found between the value indicated on the pain scale and body temperature at T0 (*P* = 0.221; *R*^2 ^= 0.009), nor at T60 (*P* = 0.391; *R*^2 ^= 0.004). By contrast, a significant correlation was observed between pain scale values at T0 and T60 (*P* = 0.008; *R*^2 ^= 0.040).

No significant differences were observed between body temperature in the various age groups either at T0 (*P* = 0.978) or T60 (*P* = 0.227) or in relation to the level of discomfort at T0 (*P* = 0.755) and T60 (*P* = 0.966) (Supplementary Appendix S6).

Similarly, no difference was observed according to the level of discomfort in the various age groups at T0 and T60 when assessing the number of altered items (*P* = 0.592 and *P* = 0.356) (Supplementary Appendix S7).

*Analysis of the subgroup of children with SARS-CoV-2 infection*

Seven (4.1%) children with SARS-Cov-2 infection (*n* = 5) or SARS-Cov-2 related pediatric multisystem inflammation syndrome (PIMPS) (*n* = 2) were included in the study. Demographic and clinical characteristics are shown in Supplementary Appendix S8. The children were all without fever after one hour and there was a significant reduction in body temperature (median: 1.8; IQR: 1.4–2.3; *P* < 0.0001) and level of discomfort (median number of altered items at T0: 10, IQR:7–15; at T60: 3; IQR:1–7; *P* < 0.001) between T0 and T60.

## 4. Discussion

This prospective observational study included 172 children admitted to a pediatric emergency department for fever and receiving paracetamol. Substantial decrease in discomfort between T0 and T60 after administration of antipyretic was documented.

The drug was well tolerated and no adverse reactions to paracetamol were observed.

In agreement with the data found in literature, the results show a significant reduction in body temperature one hour after administration of the drug and the absence of fever in almost all the children included in the study (9, 10).

As expected, according to previous literature reports (9, 10), 9% of the children still had a feverish body temperature at T60, and this was more frequent in children whose initial body temperature was very high upon admission.

The assessment of discomfort was carried out using the grid of items drawn up and previously published (3) and a semi-quantitative scale that allowed the detection of significant changes in discomfort at T0 and T60. In fact, at T0 85% of the children presented severe discomfort with more than 8 altered items, after 60 min, a reduction in the proportion of children with severe discomfort to 14% was observed. It is interesting to note that the pain was also reduced: most of the children had a value of 6 or more on the pain scale at T0 (96%), while the percentage fell to 22% at T60.

The level of discomfort was not related to the age of the child, nor to the degree of body temperature. The proportion of children with severe discomfort was similar in children under and over 24 months of age at T0 (81% vs. 96%) and T60 (16% vs. 19%). Interestingly, the grade of discomfort and pain were independent of body temperature values.

Seven children infected with SARS-CoV-2 and/or PIMPS were also included in the study. They presented outcomes similar to those observed in the remaining study population. However, the small number of cases does not allow us to draw conclusions on this issue.

Previous studies aimed at assessing the effectiveness of antipyretics are poor and rely mainly on detecting changes in body temperature or, more rarely, in the level of pain, before and after administration of antipyretics, rather than evaluating changes in the degree of discomfort (9–11). Other authors used only a semi-quantitative scoring scale, based exclusively on few items such as irritability (4), while in one randomized controlled trial published by Hay et al. in 2008 (12) a more composite criterion was used to determine discomfort in the feverish child, assessing a limited number of items, such as reduced activity, reduced appetite, and disturbed sleep. Similarly, one 1991 study by Kramer et al. included the assessment of few items such as mood, comfort, appetite, or fluid intake (6). A larger 2017 observational study by Corrard et al., including 200 children with fever and 200 afebrile children, assessed the degree of discomfort on the basis of eight behavioral items and found that the degree of discomfort was not associated with the degree of body temperature (7). In fact, some children continued to play and carry out other activities as usual or showed only moderate discomfort even at very high body temperatures, while others presented with severe discomfort even with a moderate rise in temperature (7). Our study used a more completed criterion, drawn up previously by a panel of experts (3), that included variations of sleep-wake cycle, appetite, motor activity, mood, daily habits, and facial expression.

In most of these studies, a reduction in the proportion of children with discomfort was observed after administration of antipyretics (paracetamol and/or ibuprofen, alone or alternated), although calculated using different methods and at variable times (few hours (4, 7, 12, 13), one day (9), or two days (7, 12)).

In three studies the proportion of children with discomfort relief ranged from 35%–38% (4, 9, 12), while in our study the proportion is higher (71% at T60).

The change in the level of pain was assessed in three studies, in two of which (9, 13) the action of paracetamol and ibuprofen or a combination of the two was compared, while in the third study (11) the action of the individual antipyretics alone was assessed. A reduction in the scores for the level of pain was observed in all three studies (9–11). In our study using only paracetamol as antipyretics, a reduction in the level of pain was observed too, in fact at T60 37% of children shown a value of 0–2 in the pain scale.

The independence of discomfort from fever suggests that they may be expressions of two different metabolic paths that are activated simultaneously during fever, as previously speculated by other authors (7). Considering that the main aim of administering antipyretics is to reduce discomfort, our results show of a substantial reduction in the level of discomfort suffered by the child.

*Our study has some limitations.* The assessment was carried out both by directly observing the child and by interviewing the parents and, depending on their age, the child. The presence of a greater or lesser degree of fever-phobia in the observer and guardians may have influenced the results. This may be even more true considering the fact that the study was conducted in a pediatric emergency room. This may have amplified the parents' fever-phobia and the child may have been more stressed than at home. Consequently, it was decided to not interview the children if they were subjected to invasive or disturbing procedures, such as taking blood samples, administering inhalation therapy by aerosol, intravenous, oral or intramuscular therapy, applying dressings, x-rays or other similar interventions. The presence of fever-phobia in the parents, along with the hospital setting, should not have influenced the change in discomfort before and after administration of paracetamol.

Pain was assessed using the FPS-R (5) scale, by asking the children to indicate the facial expression that best represents their level of pain. This scale has been validated for self-assessment of pain from the age of 4 years and it is considered a highly reliable and reproducible indicator of pain (14). The same scale was used in children under 3 years of age, asking parents/tutors to identify the facial expression that most closely resembled that of the child at the time of the fever. This approach is similar to that one previously adopted by other authors (7).

In conclusion, the study is one of the few available in literature to have assessed the alteration of discomfort in parallel with the reduction of body temperature in pediatric patients. The data confirms the recommendations of national and international guidelines in relation to the use of antipyretics in children with discomfort in order to reduce their level of general discomfort.

## Data Availability

The raw data supporting the conclusions of this article will be made available by the authors, without undue reservation.
