# Supplementary material for: Discomfort relief after paracetamol administration in febrile children admitted to a third level paediatric emergency department
Source: Front Pediatr. 2023 Mar 9;11:1075449. doi: 10.3389/fped.2023.1075449 (PMC10034175; doi:10.3389/fped.2023.1075449)
Supplement: Supplementary file 1 [file Datasheet1.pdf]

Appendix 1: Grid used for the assessment of every child included in the study.

Patient's code	Ethnic group
Age	Place of birth
Sex	Origins of parents
Breath frequency	Admission diagnosis
Heart rate	Discharge dimission
Drugs administrered in the prevous 12 hours	
Body temperature at the entrance	Type of thermomiter
Body temperature after 1 hour	

	Admission			After 1 hour		
	Less	As usual	More	Less	As usual	More
**Variations of Sleep-Wake Rhythm**						
Delayed sleep phase						
Early sleep phase						
Night awakenings						
**Appetite Variations**						
Reduced appetite						
Reduced liquid intake						
**Variation in Motor Activity**						
Increased complain						
Restlessness						
Weeknes						
Fatigue						
**Change in Mood**						
Irritability						
Anger						
Weeping						
**Variation in Daily Habits**						
Play reduction						
Reduction in showing interests						
Seeking comfort						
Uncooperative						
**Variation of Facial Expression**						
Changed Look						
Clenched teeth						
Curled lips						
Wrinkled forehead						
Paleness/colour change						
**Other Signals**						
Tachypnoea						
Chills						
Widespread pain						

Type of antipyretic	
Posology	
Method of administration (Dose/kg)	Weight
Vomit in the following hour	
Defecation after rectal administration	
Other drugs	
Other therapeutic or diagnostic maneuvers	

Which expression reflects more the one of the child at the entrance?

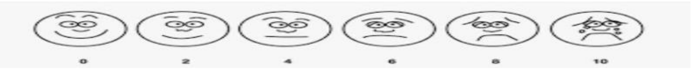

Which expression reflects more the one of the child after 1 hour?

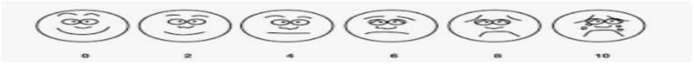

Appendix 2: comparison of demographic and clinical characteristics of children with severe and less severe discomfort.

Characteristic	Important Dicomfort	Not important discomfort	P
**Age (n;%)**			
0-24	15 (62,5%)	94 (63,5%)	0,9
25-36	1 (4,2%)	12 (8,2%)	
>37	8 (33.3%)	42 (28,4%)	
**Sex (n;%)**			
F	10 (41,7%)	71(48,0%)	0,6
M	14 (58,3%)	77(52,0%)	
**Ethnic group (n;%)**			
Caucasian	22 (91,7%)	126 (85,2%)	0,4
Afro-American	1 (4,2%)	6 (4,1%)	
Asian	1 (4,2%)	6 (4,1%)	
South-American	0 (0,0%)	10 (6,8%)	
**Body temperature at T0 (°C)**			
Median °C	39	38,8	0,4
IQR °C	38,5-39,9	38,3-39,3	
**Body temperature at T60 (°C)**			
Median °C	36,8	36,8	0,3
IQR °C	36,3-37,3	36,4-37,5	
**Variation of body temperature**			
Median °C	1,9	2	0,2
IQR °C	1,1-2,5	1,5-2,4	
**Children with/without fever (n;%)**			
Without fever	20 (83,3%)	137 (92,6%)	0,1
With fever	4 (16,7%)	11 (7,4%)	
**Admission diagnosis (n:%)**			
Fever	18 (75%)	120 (81,1%)	0,5

Abdominal pain	3 (12,5%)	7 (4,7%)	
Convulsion	1 (4,2%)	6 (4,1%)	
Nausea/vomit	2 (8,3%)	5 (3,4%)	
Headache	0 (0,0%)	3 (2,0%)	
Dyspnea	0 (0,0%)	3 (2,0%)	
ORL disorder	0 (0,0%)	1 (0,7%)	
Ophtalmic disorder	0 (0,0%)	1 (0,7%)	
Suspected PIMS	0 (0,0%)	1 (0,7%)	
Stomatitis	0 (0,0%)	1 (0,7%)	
**Discharge dimission (n;%)**			
Viral infection/Infection of the upper airways	12 (45,8%)	84 (51,4%)	0,5
Urinary tract infection	2 (8,3%)	15 (10,1%)	
Convulsion	1 (4,2%)	7 (4,7%)	
Gastroenteritis	2 (13,4%)	7 (4,8%)	
Acute otitis media	1 (4,2%)	6 (4,1%)	
Appendicopathy	0 (0,0%)	4 (2,7%)	
SARS-CoV-2 infection	2 (8,3%)	4 (2,7%)	
Mesenteric Lymphadenitis	1 (4,2%)	3 (2,0%)	
Stomatis	0 (0,0%)	3 (2,0%)	
Perianal abscesses	0 (0,0%)	2 (1,4%)	
Abdominal pain	1 (4,2%)	1 (0,7%)	
Pneumonia	0 (0,0%)	3 (5,1%)	
PIMS	0 (0,0%)	2 (1,4%)	
Athralgia	0 (0,0%)	1 (0,7%)	
Bronchiolitis	0 (0,0%)	1 (0,7%)	
Heahache	0 (0,0%)	1 (0,7%)	
Feet cellulitis	0 (0,0%)	1 (0,7%)	
Conjiuntivitis	0 (0,0%)	1 (0,7%)	
Intussusception	0 (0,0%)	1 (0,7%)	
Esophageal duplication	0 (0,0%)	1 (0,7%)	

Encefalitis	0 (0,0%)	1 (0,7%)	
Stranguria	0 (0,0%)	1 (0,7%)	
**Outcome (n;%)**			
Discharge	15 (62,5%)	86 (58,1%)	0,7
Brief intensive observation (BIO)	5 (20,8%)	31 (20,9%)	
Hospitalisation	4 (16,7%)	31 (20,9%)	

Appendix 3: comparison between demographic and clinical characteristics of children with and without fever.

Characteristic	Without fever	With fever	P
**Age (n;%)**			
0-24	100 (63,7%)	9 (60%)	0,8
25-36	10 (6,4%)	3 (20%)	
>37	47 (29,9%)	3 (20%)	
**Sex (n; %)**			
F	74 (47,1%)	7 (46,7%)	0,9
M	83 (52,9%)	8 (53,3%)	
**Ethnic group (n; %)**			
Caucasian	138 (87,9%)	10 (66,7%)	0,02
Afro-American	5 (3,2%)	1 (6,7%)	
Asian	5 (3,2%)	2 (13,3%)	
South-American	9 (5,7%)	2 (13,2%)	
**Body temperature at T0 (°C)**			
Median °C	38,7	39,9	<0,001
IQR °C	38,3-38,1	39,3-40	
**Body temperature at T60 (°C)**			
Median °C	36,8	38	
IQR °C	36,3-37,3	38-38,3	
**Variation of body temperature**			
Median °C	2	1,5	0,012
IQR °C	1,5-2,5	1,3-2,0	
**Classes of discomfort T0 (n;%)**			
0	0 (0,0%)	1 (6,7%)	
1-4	2 (1,3%)	0 (0.0%)	
5-8	18(11,5%)	5 (33,3%)	0,02
9-12	51 (32,5%)	2 (13,3%)	
>13	86 (54,8%)	7 (46,7%)	0,5

**Classes of discomfort T60 (n;%)**			
0	34 (21,7%)	4 (26,7%)	
1-4	63 (40,1%)	6 (40,0%)	0,9
5-8	36 (22,9%)	5 (33,3%)	0,4
9-12	14 (8,9%)	0 (0.0%)	
>13	10 (6,4%)	0 (0.0%)	
**Admission diagnosis (n:%)**			
Fever	124 (78,9%)	14 (93,3%)	0,2
Abdominal pain	9 (5,8%)	1 (6,7%)	
Convulsion	7 (4,5%)	0 (0,0%)	
Nausea/vomit	7 (4,4,%)	0 (0,0%)	
Headache	3 (1,9%)	0 (0,0%)	
Dyspnea	3 (1,9%)	0 (0,0%)	
ORL disorder	1 (1,9%)	0 (0,0%)	
Ophtalmic disorder	1 (0,6%)	0 (0,0%)	
Suspected PIMS	1 (0,6%)	0 (0,0%)	
Stomatitis	1 (0,6%)	0 (0,0%)	
**Discharge dimission (n;%)**			
Viral infection/Infection of the upper airways	85 (50,8%)	10 (70,5%)	0,3
Urinary tract infection	16 (10,2%)	1 (6,7%)	
Convulsion	7 (4,5%)	1 (6,7%)	
Gastroenteritis	7 (4,5%)	2 (13,4%)	
Acute otitis media	7 (4,5%)	0 (0,0%)	
Appendicopathy	6 (3,8%)	0 (0,0%)	
SARS-CoV-2 infection	5 (3,2%)	0 (0,0%)	
Mesenteric Lymphadenitis	3 (1,9%)	0 (0,0%)	
Stomatis	2 (1,3%)	1 (6,7%)	
Perianal abscesses	2 (1,3%)	0 (0,0%)	
Abdominal pain	2 (1,3%)	0 (0,0%)	

Pneumonia	8 (1,9%)	0 (0,0%)	
PIMS	2 (1,3%)	0 (0,0%)	
Athralgia	1 (0,6%)	0 (0,0%)	
Bronchiolitis	1 (0,6%)	0 (0,0%)	
Heahache	1 (0,6%)	0 (0,0%)	
Feet cellulitis	1 (0,6%)	0 (0,0%)	
Conjiuntivitis	1 (0,6%)	0 (0,0%)	
Intussusception	1 (0,6%)	0 (0,0%)	
Esophageal duplication	1 (0,6%)	0 (0,0%)	
Encefalitis	1 (0,6%)	0 (0,0%)	
Stranguria	1 (0,6%)	0 (0,0%)	
**Outcome (n;%)**			
Discharge	95 (60,5%)	6 (40,0%)	0,1
Brief intensive observation (BIO)	31 (19,7%)	5 (33,3%)	
Hospitalisation	31 (19,7%)	4 (26,7%)	

Appendix 4: correlation between a) altered items at T0 and T60, b) body temperature at T0 and T60, c) altered items and body temperature at T0, d) altered items and body temperature at T60, e) difference between number of altered items at T0 and T60 and temperature difference at T0 and T60.

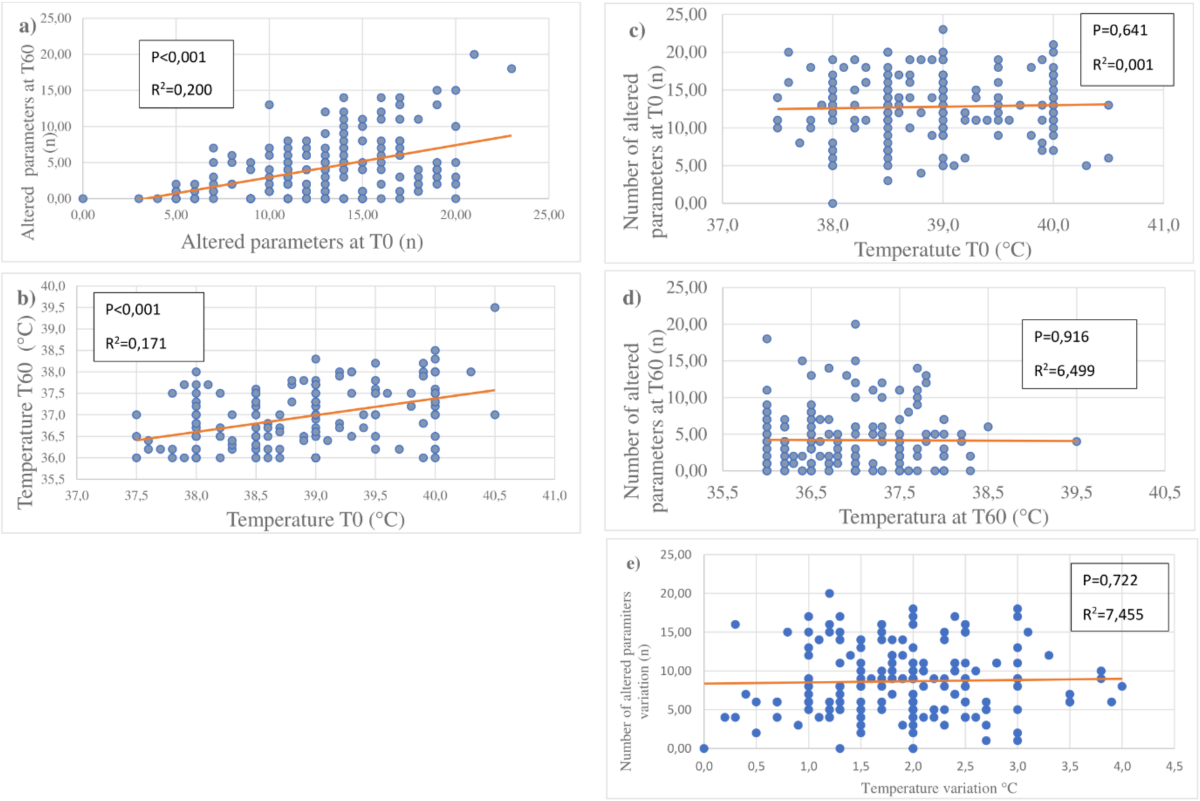

Appendix 5: correlation between, a) pain scale and body temperature at T0, b) pain scale and body temperature at T60 c) pain scale at T0 and T60

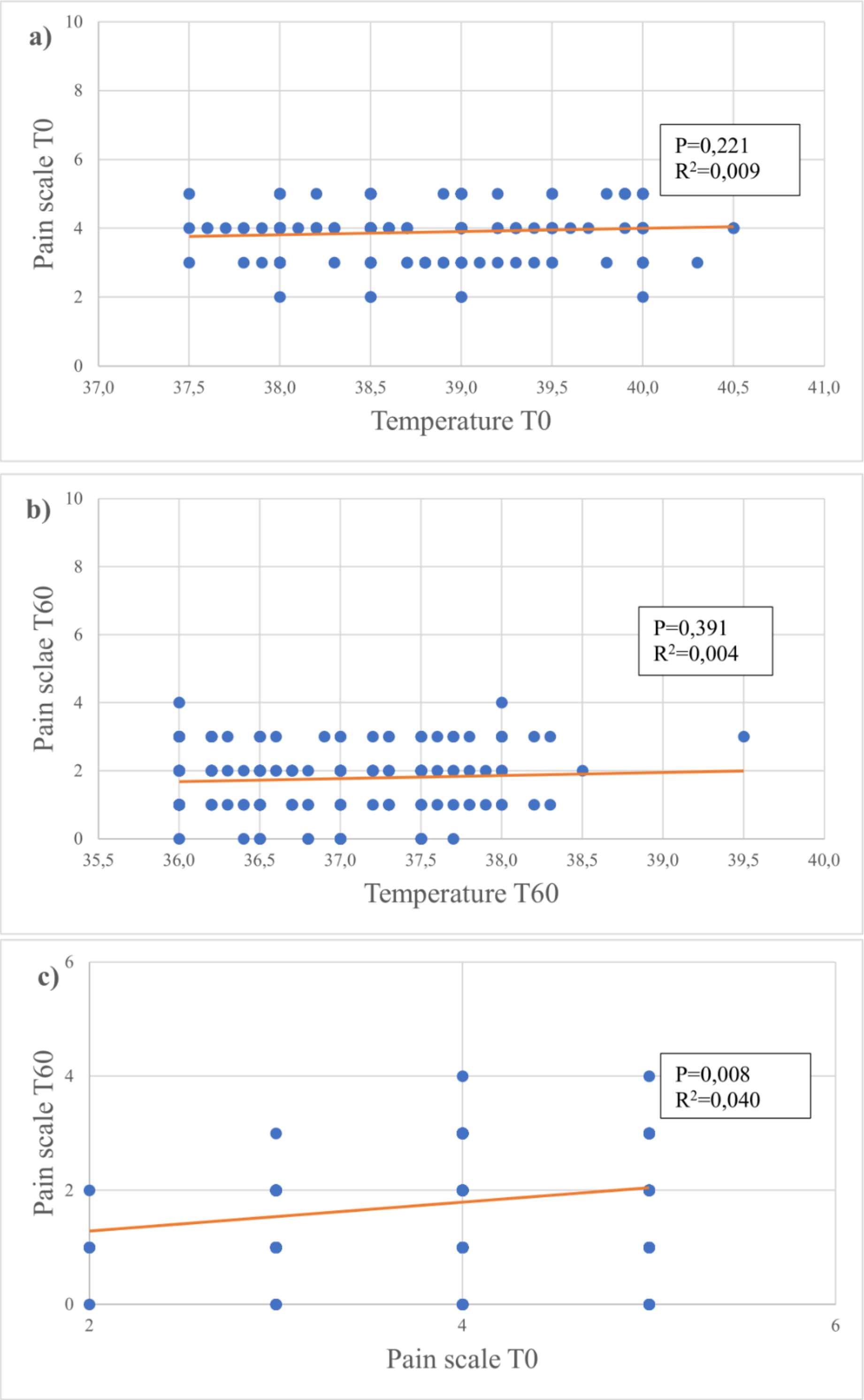

Appendix 6: a) correlation between age groups and a) body temperature at T0 and T60, b) level of discomfort (expressed as the number of altered items) at T0 and T60.

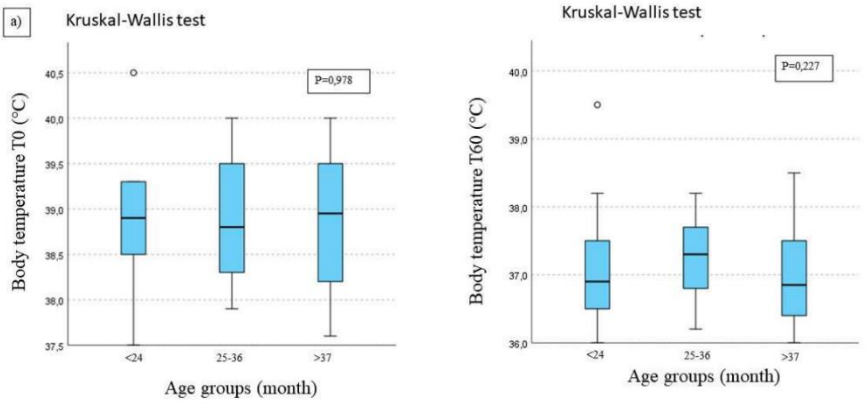

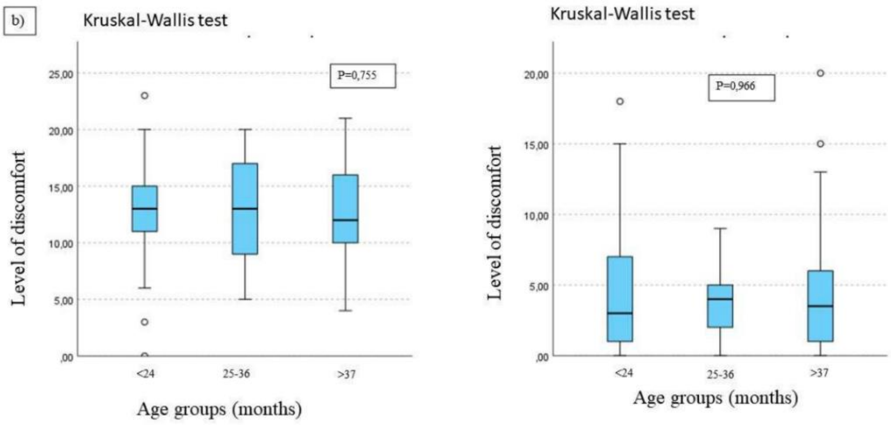

Appendix 7: correlation between classes of altered items and body temperature at T0 and T60.

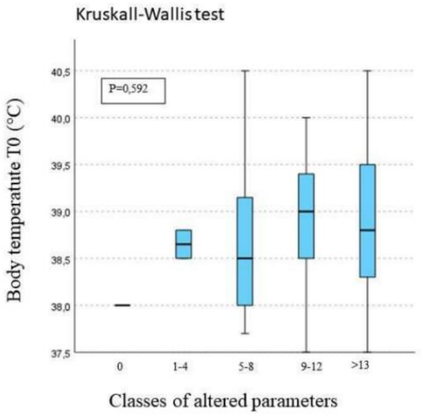

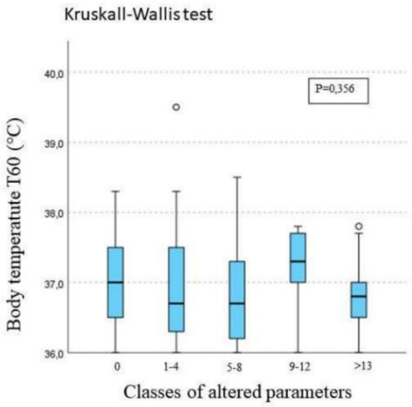

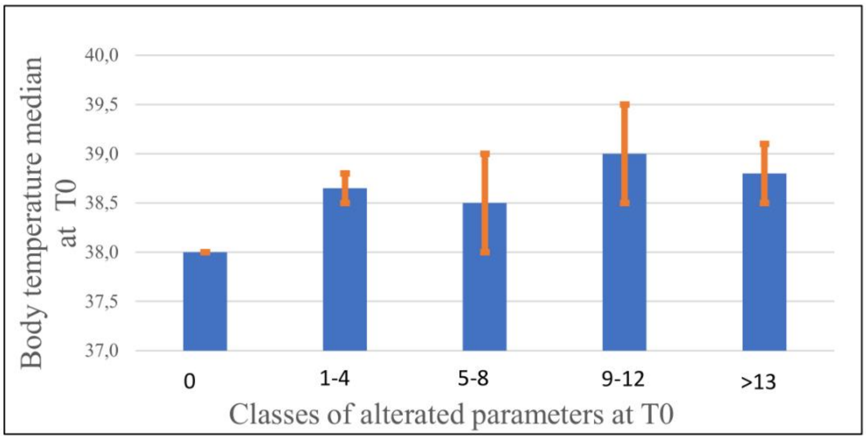

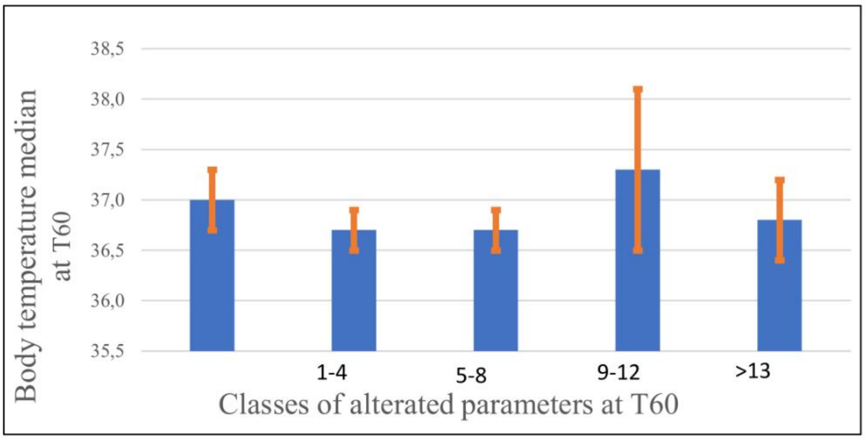

Appendix 8: demographic and clinical characteristics of children with SARS-Cov-2 and PIMS infection

Characteristic	Children	
**Sex (n; %)**		
M	3	43
F	4	57
**Ege (months)**		
0-24	6	86
25-36	0	0
>37	1	14
Median	1	
IQR	0,5-3,5	
**Ethnic group (n;%)**		
Caucasian	6	86
Afro-American	0	0
Asian	1	14
South-American	0	0
**Body temperature at T0 °C**		
Median (°C)	38,2	
IQR (°C)	38,1-38,8	
**Body temperature at T60°C**		
Median (°C)	36,5	
IQR (°C)	36,0-37,4	
**Variation of body temperature °C**		
Median	1,8	
IQR	1,4-2,3	
**Class of discomfort at T0 (n; %)**		
0	0	0
1-4	0	0
5-8	2	29

9-12	2	29
>13	3	43
**Class of discomfort at T60 (n; %)**		
0	0	0
1-4	5	71
5-8	2	29
9-12	0	0
>13	0	0
**Children with/without fever (n;%)**		
Without fever	7	100%
With fever	0	0%
**Admission diagnosis (n; %)**		
Fever	5	71
Suspected PIMS	2	29
**Discharge dimission (n; %)**		
PIMS	2	29
Sars-Cov-2 infection	5	71
**Outcome (n; %)**		
Discharge	2	29
Brief intensive observation (BIO)	0	0
Hospitalisation	5	71
